# Spin–Flop and Metamagnetic Transition in Monoclinic Eu_4_Bi_6_Se_13_

**DOI:** 10.1021/acs.chemmater.4c03185

**Published:** 2025-02-21

**Authors:** Mingyu Xu, Jose L. Gonzalez Jimenez, Greeshma C. Jose, Artittaya Boonkird, Chengkun Xing, Chelsea Harrod, Xinle Li, Haidong D. Zhou, Xianglin Ke, Wenli Bi, Mingda Li, Weiwei Xie

**Affiliations:** † Department of Chemistry, Michigan State University, East Lansing, Michigan 48864, United States; ‡ Department of Physics, University of Alabama, Birmingham, Alabama 35294, United States; § Quantum Measurement Group, Massachusetts Institute of Technology, Cambridge, Massachusetts 02139, United States; ∥ Department of Nuclear Science and Engineering, Massachusetts Institute of Technology, Cambridge, Massachusetts 02139, United States; ⊥ Department of Physics, University of Tennessee, Knoxville, Tennessee 37996, United States; # Department of Chemistry, Clark Atlanta University, Atlanta, Georgia 30314, United States; ∇ Department of Physics and Astronomy, Michigan State University, East Lansing, Michigan 48864, United States

## Abstract

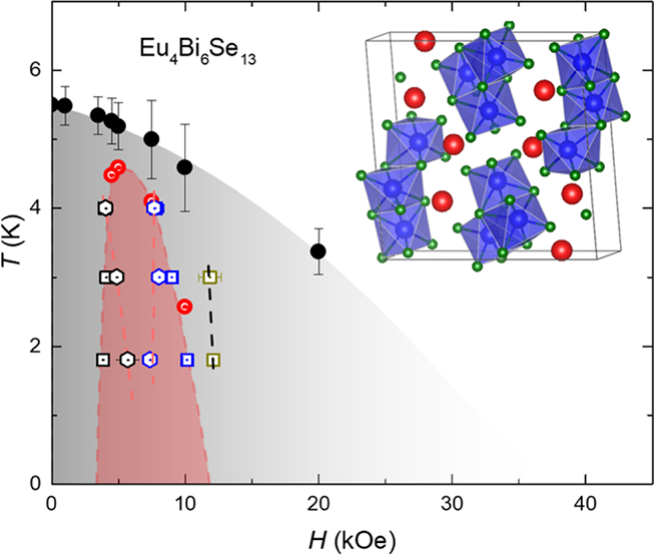

This study explores an investigation of the crystallographic, electronic, and magnetic properties of the europium-based bismuth selenide compound Eu_4_Bi_6_Se_13_, with particular focus on its magnetic anisotropy. This compound adopts a monoclinic crystal structure classified under the *P*2_1_/*m* space group (#11). It exhibits distinctive structural features, including substantial Eu–Se coordination numbers (6 and 8), Bi–Se ladders, and linear chains of Eu atoms that propagate along the *b*-axis. Electronic resistivity assessments indicate that Eu_4_Bi_6_Se_13_ exhibits metallic behavior. As the magnetic field is oriented along the *b*-axis, magnetic characterization reveals uniaxial magnetic anisotropy, with metamagnetic transitions appearing at approximately 12 kOe and a lower field. In the field below 10 kOe, the spin-flop transition is observed with possible domain-induced hysteresis. This behavior supports the identification of metamagnetic features in field-dependent measurements attributable to the europium spins.

## Introduction

Magnetic topological materials represent a burgeoning field of research that bridges the gap between topological quantum states and magnetism. These materials, characterized by their intrinsic magnetic orders and nontrivial topological electronic structures, have garnered significant attention for their potential to realize exotic quantum phenomena such as the quantum anomalous Hall effect, axion insulators, and Majorana fermions.^1−8^ In europium (Eu)-based magnetic topological materials, Eu^2+^, with its strong magnetic moments (4*f*^7^), plays a pivotal role in inducing and tuning the magnetic interactions within these compounds, thereby influencing their topological properties. The interaction between the localized magnetic moments of europium atoms and the itinerant electrons from other atoms in the crystal lattice can lead to a variety of magnetic ground states, including ferromagnetic, antiferromagnetic, and more complex configurations.^9^ Numerous Eu-based ternary compounds have been identified to display magnetic and topological characteristics, predominantly incorporating pnictogens. This category includes families such as EuMnSb_2_^10,11^ and EuCd_2_As_2_,^12−14^ where the structured alternation of magnetic and nonmagnetic layers plays a pivotal role in modulating their electronic properties. For example, the observation of anomalous negative magnetoresistance in the topological semimetal EuMg_2_Bi_2_^15,16^ and the topological insulator EuIn_2_As_2_ is attributed to the strength of ferromagnetic interactions manifested within A-type antiferromagnetic ordering. However, very little research has been done to explore the electronic and magnetic properties in novel ternary phases containing chalcogenides and Eu^2+^. Within this realm of investigation, the MnBi_2_*X*_4_ (*X* = Se, Te) systems have attracted significant interest owing to emergent topological phases intimately associated with their layered antiferromagnetic ordering.^17−23^ This prompts an intriguing line of inquiry regarding the substitution of Mn^2+^ with Eu^2+^, aiming to explore the potential emergence of novel phases. Such a substitution could potentially alter the magnetic and electronic landscapes of these compounds, providing fertile ground for the discovery of new quantum states influenced by the unique magnetic characteristics of Eu^2+^.

Thus, in this study, we report the synthesis of a europium bismuth chalcogenide, Eu_4_Bi_6_Se_13_, which exhibits low-field metamagnetic behavior along the crystalline *b*-axis and retains metallic conductivity throughout the entire temperature regime. This compound represents the first instance within the chalcogenide family—predominantly characterized by orthorhombic space group symmetry^24−26^—to crystallize in a monoclinic unit cell. Moreover, the temperature- and field-dependent magnetic studies demonstrate that spin-flop and metamagnetic transitions occur at applied magnetic fields. This observation expands the structural diversity observed in europium bismuth chalcogenides and underscores the unique magnetic and electronic phenomena inherent to this material.

## Experimental Methods

### Crystal Growth

Needle-shaped crystals of Eu_4_Bi_6_Se_13_ were synthesized by utilizing a solid-state reaction technique. An ingot of Europium (sublimed dendritic, REO grade, sourced from Thermo Scientific), finely ground Bismuth pieces (purity of 99.99%, provided by Strem Chemicals Inc.), and Selenium powder (with a purity of 99.995%, obtained from Thermo Scientific) in a 4:6:13 ratio were homogeneously mixed and pressed into pellets, each weighing about 1000 mg. These pellets were placed in an alumina crucible and sealed in evacuated quartz tubes (with a vacuum level maintained below 1 × 10^–5^ Torr) to prevent oxidation and contamination. The ampule was heated to 800 °C at a ramp rate of 60 °C per hour, maintained at 800 °C for a duration of 48 h to ensure a complete reaction, and then cooled down at the same rate to room temperature. The needle-shaped single-crystalline Eu_4_Bi_6_Se_13_ was obtained with extra EuSe, Bi-alloys, and Se-alloys phases. Since the shape of Eu_4_Bi_6_Se_13_ is needle-like, it can be mechanically separated from the other phases. The separated pure Eu_4_Bi_6_Se_13_ crystals are used in all the measurements.

### Structural and Chemical Composition Determination

The single crystal X-ray diffraction of Eu_4_Bi_6_Se_13_ was taken using a Rigaku XtaLab Synergy-S X-ray diffractometer equipped with Mo radiation (λ_Kα_ = 0.71073 Å) and an Oxford Cryosystems 800 low-temperature device. The selected pure-phase single crystal was mounted on a nylon loop with PARATONE oil. To minimize data acquisition time and prevent the excessive accumulation of peaks arising from the low-symmetry monoclinic cell, measurements were carried out at 100 K, with an exposure duration of 5.0 s per frame. The total number of runs and images was based on the strategy calculation from the program CrysAlisPro 1.171.43.92a (Rigaku OD, 2023). Data reduction was performed with correction for Lorentz polarization.^27^ Moreover, an empirical absorption correction employing spherical harmonics was applied within the SCALE3 ABSPACK scaling algorithm to refine the data further.^28^ The structure was solved and refined using the SHELXTL Software Package.^29,30^

The sample phase composition was analyzed by employing a JEOL 6610LV scanning electron microscope equipped with a tungsten hairpin emitter (JEOL Ltd., Tokyo, Japan). For elemental analysis, energy-dispersive X-ray spectroscopy (EDX) was conducted utilizing an Oxford Instruments AZtec system (Oxford Instruments, High Wycombe, Buckinghamshire, England), operating software version 3.1. This setup included a 20 mm^2^ silicon drift detector (SDD) and an ultrathin window integrated with the JEOL 6610LV SEM. The needle-like crystals were affixed to carbon adhesive tape and introduced into the SEM chamber for examination at an accelerating voltage of 20 kV. Data acquisition entailed collecting spectra at multiple points along the individual crystals over an optimized time frame. Quantitative compositional analysis was performed using SEM Quant software, which applied corrections for matrix effects to the intensity measurements.

### Phase Identification

The temperature-dependent PXRD patterns were measured using a HUBER X-ray diffractometer with Cu Kα radiation (λ = 1.5460 Å), equipped with a helium cryogenic system. A step size of 0.005° was used to measure spectra over a Bragg angle (2θ) range of 4–100°. The powdered sample was measured every 10 K from 300 to 10 K. The powder data were refined through the Rietveld method using the GSASII software.

### Magnetic and Electronic Properties Measurements

The temperature- and field-dependent VSM magnetization measurements and resistance measurements, as well as the temperature-dependent specific heat of Eu_4_Bi_6_Se_13_ single crystals, were carried out using a Quantum Design Physical Property Measurement System (PPMS-DynaCool). In temperature- and field-dependent magnetization, the needle-shaped single crystal samples were arranged carefully in parallel orientation on Kapton tape and secured on a quartz paddle sample holder. The magnetic signal from the tape is not considered. Around 1 mg samples were measured parallel and perpendicular to the *b*-axis with a magnetic field up to 90 kOe. DC electrical resistance measurements were performed in a standard four-contact geometry with a 1 mA current. 50 μm diameter Pt wires were bonded to the samples with silver conductive epoxy covered by silver paint (DuPont 4929N) with contact resistance values of about 2–3 Ω. Up to 90 kOe, the magnetic field was applied perpendicular to the *b*-axis, with the current flowing parallel to the *b-*axis. Temperature-dependent specific heat measurements were carried out using the relaxation technique as implemented in the Heat Capacity option of the PPMS.

## Results and Discussion

**Figure 1:**
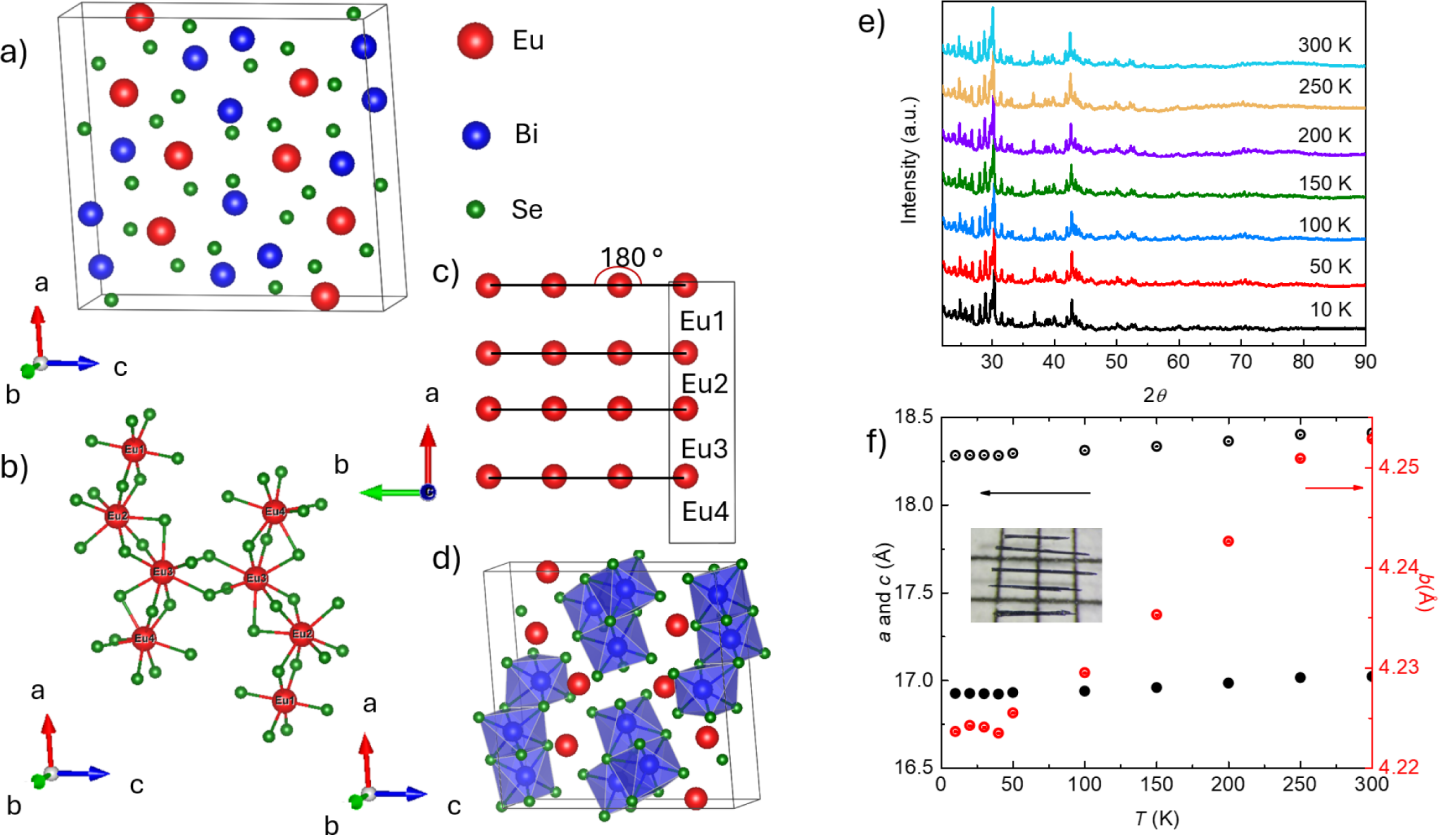
Crystal structure of Eu_4_Bi_6_Se_13_and temperature-dependent powder X-ray diffraction patterns. The structure of Eu_4_Bi_6_Se_13_ is shown in (a). The black line gives the unit cell. (b) The coordination environment of each Eu site. The linear chains of Eu–Eu interactions extending along the *b*-axis are shown in (c). (d) The edge-share distorted BiSe_6_ octahedra. (e) Powder X-ray diffraction (PXRD) data at various temperatures from 300 to 10 K. (f) The lattice parameters refined from PXRD. The inset shows a picture of crystals on the millimeter grid paper.

Eu_4_Bi_6_Se_13_ crystallized with the monoclinic *P*2_1_/*m* space group, exhibiting isostructural properties with Sr_4_Bi_6_Se_13_.^31^Figure 1a shows the crystal structure obtained from single-crystal X-ray diffraction refinement (SCXRD data shown in Tables S1 and S2). As shown in Figure 1b, four different europium sites are surrounded by bismuth sites. To the best of our understanding, this represents the inaugural instance of an Eu–Bi–Se adopting a monoclinic framework. Chemically analogous entities typically assume an orthorhombic architecture yet display congruent unit cell dimensions and structural motifs as identified in this novel configuration. As illustrated in Figure 1b, such motifs include relatively planar cells punctuated by Bi–Se connectivity, in either ladder or columnar arrangements, alongside extensive Eu^2+^ coordination environments. Notably, the coordination geometries around the Eu^2+^ sites vary within the structure. The Eu1 atom is encased in a 6-fold coordination by Se atoms, constituting a distorted octahedral geometry with an average bond length of 3.098 (1) Å. In contrast, Eu2 and Eu4 atoms engage in an 8-fold coordination, forming a distorted square antiprism with a singular square face apparent. The Eu3 atom, uniquely, is nine-coordinated, adopting elongated bond lengths to maintain its divalent state, evidenced by an average bond length of 3.321 (1) Å. As depicted in Figure 1c, Eu–Eu interactions yield linear chains extending along the *b*-axis, characterized by a consistent bond distance that aligns with a unit cell length of 4.219 (1) Å. Figure 1d presents the edge-shared distorted BiSe_6_ octahedra, forming the octahedra chains and blocks. The Eu atoms are embedded in layers of the octahedra. Phase purity was ascertained through powder LeBail refinement employing X-ray data acquired at various temperatures from 300 to 10 K, as illustrated in Figure 1e. Figure 1f gives the *a*, *b*, and *c* lattice parameters at different temperatures, refined by PXRD data; the volume and β change are shown in Figure S4, compared with ambient pressure SCXRD data summarized in Tables S1 and S2. Phase composition was further validated through SEM-EDS analysis, as depicted in Figure S1.

**Figure 2:**
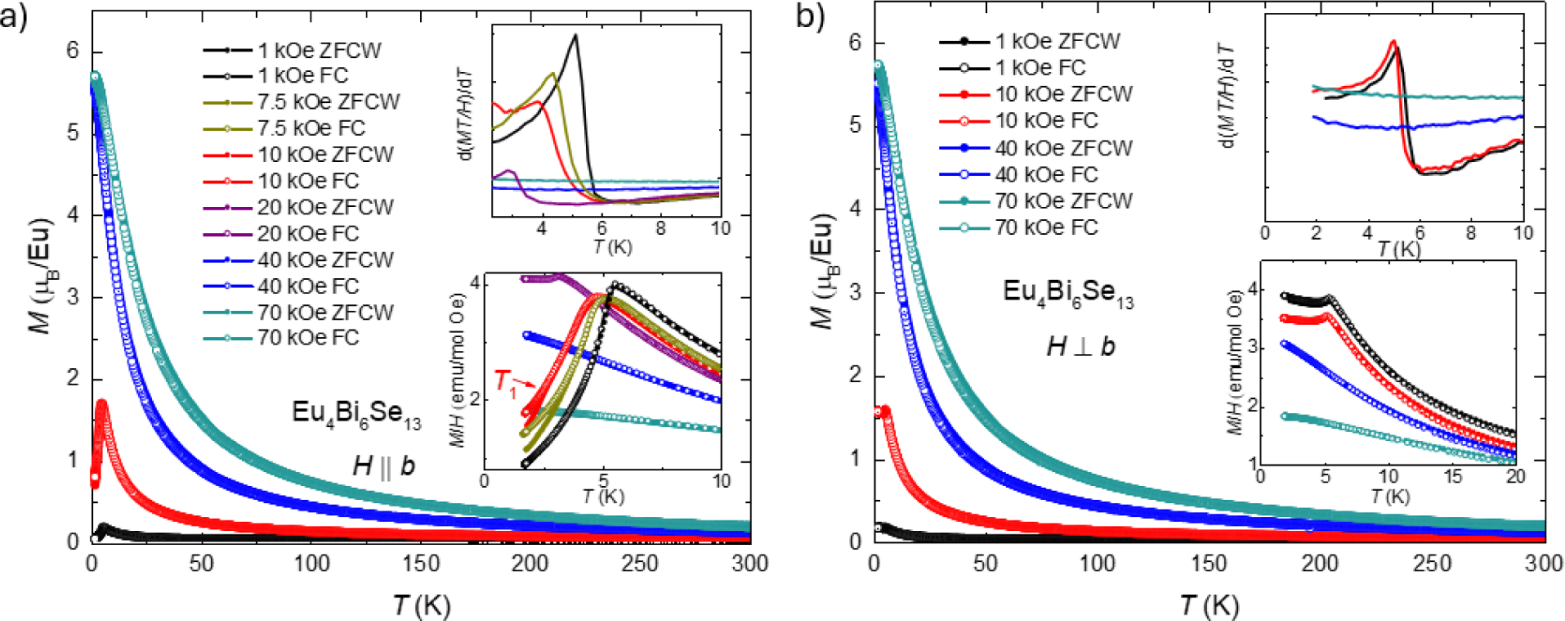
Temperature-dependent magnetization in the different magnetic fields. Temperature-dependent magnetization in the different magnetic fields parallel (a) or perpendicular (b) to the *b*-axis is shown with zero-field-cool-warming (ZFCW) and field-cool (FC) temperature protocols. The lower insets show the magnetization at a low-temperature range. The upper insets present the temperature derivative of the value *M* × *T*/*H*.^32^*M* presents magnetization, *T* presents temperature, and *H* presents magnetic field; *T*_1_ gives the temperature at which the ZFCW and FC magnetization split. The criterion of *T*_1_ is shown in the inset of Figure S2a.

Figure 2a,b presents the temperature-dependent magnetization with the magnetic field parallel or perpendicular to the *b*-axis, respectively, using zero-field-cool-warming (ZFCW) and field-cool (FC) temperature protocols. There is no observable difference between ZFCW and FC measurements at temperatures from 5 to 300 K with magnetic fields from 1 kOe to 70 kOe. All the magnetization shows tail-like behavior as a function of temperature before a feature appears in the low magnetic field around 5 K. This feature shows a sudden decrease in magnetization, indicating the antiferromagnetic transition. When the magnetic field is increased up to 40 kOe, no feature is observed above 1.8 K. Compared to the feature when the field is applied perpendicular to the *b*-axis, the magnetization with the field parallel to the *b*-axis decreases by a much larger value, indicating clear anisotropy. We assume this feature indicates the phase transition based on further investigation. If we define the transition temperature using the average onset and offset values, as shown in Figure S2a, the upper insets show a suppression of the transition temperature as the magnetic field increases. The transition temperature in the field parallel to the *b*-axis suppresses much faster than when perpendicular to the *b*-axis. The lower insets show details of temperature-dependent magnetization in the low-temperature range. Except for a clear difference in the decreased value of magnetization after the transition, hysteresis is shown at a low temperature in the range of magnetic field of 1 kOe < H < 20 kOe with the magnetic field direction parallel to the *b*-axis; however, in another direction, no hysteresis is observed. The discussion above suggests that the magnetic easy axis is along the *b*-axis. There is almost no anisotropy before the transition, as shown in Figure S5.

**Figure 3:**
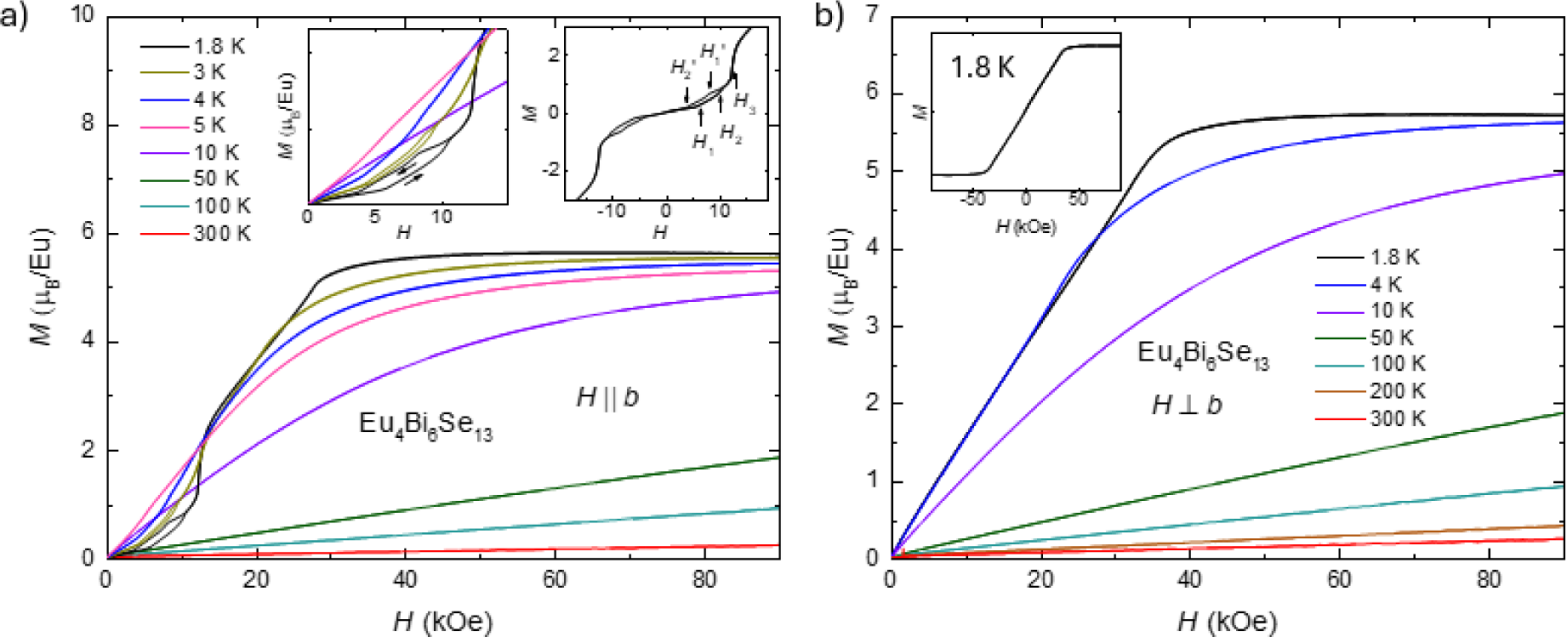
Field-dependent magnetization at different temperatures. Magnetic field-dependent magnetizations in the different magnetic fields parallel (a) or perpendicular (b) to the *b*-axis are shown. The left inset of (a) shows the details of low-field magnetization. The right inset in (a) and the inset in (b) show the full loop measurement of magnetization as a function of the magnetic field at 1.8 K with a certain field range. Measurements were taken up to 90 kOe. The arrows in the right inset of (a) indicate the direction of the magnetic fields changing. The arrows indicate *H*_1_, *H*_2_, *H*_1_’, *H*_2_’, and *H*_3_, which represent the feature fields determined by Figure S2b.

Figure 3 presents the field-dependent magnetization at different temperatures with the magnetic field parallel (Figure 3a) or perpendicular (Figure 3a) to the *b*-axis. As the magnetic field increases to 90 kOe, the magnetization is saturated when the temperature is below 5 K. When the temperature is above 50 K, linear field-dependent magnetization is observed in the field range 0–90 kOe. As the field is perpendicular to the *b*-axis, no hysteresis is shown. When the magnetic field is parallel to the *b*-axis direction, hysteresis is observed at a certain magnetic field and temperature range. At 1.8 K, no hysteresis is shown at a small field range below 3 kOe. When the field increases, the hysteresis loop appears. As the field continues to increase, the metamagnetic-like jump of magnetization occurs around 12 kOe. When the magnetic fields increase further, magnetization increases on the same slope as in the other direction before saturation. The right inset of Figure 3a gives the full loop of magnetization as a function of the magnetic field at 1.8 K with a field up to 90 kOe (to show the hysteresis clearly, the range of the magnetic field shown in the inset is from −18 kOe to 18 kOe). The saturation moment is 5.68 μ_B_/Eu when the field is parallel to the *b*-axis, which is very similar to the saturation moment of 5.72 μ_B_/Eu when the field is perpendicular to the *b*-axis. The arrows show the feature fields *H*_1_, *H*_2_, *H*_1_’, *H*_2_’, and *H*_3_, which are determined by Figure S2b. As the temperature increases, the hysteresis size becomes smaller. At 5 K, almost no hysteresis is observed. When the temperature increases to 5 K, the *H*_1_, *H*_2_, *H*_1_’, *H*_2_’, and *H*_3_ features disappear. The field perpendicular to the *b*-axis measurements shows no hysteresis or jump-like feature.

**Figure 4:**
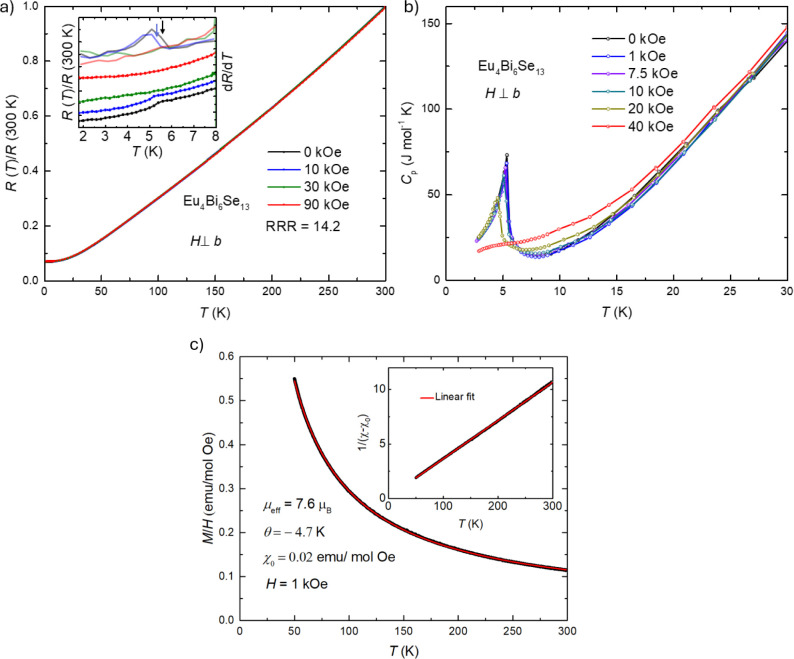
Normalized resistance and specific heat measurements. Normalized resistance and specific heat measurements were taken in different magnetic fields perpendicular to the *b*-axis. (a) Normalized resistance (*R*(*T*)/*R*(300 K)) as a function of temperature in the different magnetic fields perpendicular to the *b*-axis. The inset gives the low-temperature range of resistance (plotted with symbols and lines) and *dR*/*dT* (plotted with transparency lines). The arrows indicate the transition temperatures. The color code of the inset is the same as that in (a). (b) Temperature-dependent specific heat in different magnetic fields perpendicular to the *b*-axis. (c) Curie–Weiss (CW) fitting on the polycrystalline average susceptibility with the range of temperature 50 K–300 K on a polycrystalline average susceptibility calculated as χ = (2χ_⊥_ + χ_∥_)/3, where χ_⊥_ and χ_∥_ denote susceptibilities perpendicular and parallel to the crystallographic *b*-axis, respectively. The inset shows the linear fitting with subtract χ_0_.

Figure 4a presents the temperature-dependent resistance measurements in different magnetic fields perpendicular to the *b*-axis. The resistance as a function of temperature shows metallic behavior with an RRR (residual resistance ratio) of 14.2. Overall, in the direction of *H* ⊥ *b*, the resistance does not change much under different fields; however, as shown in the inset, kink-like features are observed at low temperature and low field, which are suppressed by a magnetic field. These features are clearer in the d*R*/d*T* plot. These feature temperatures are around 5 K and decrease with an increase in the magnetic field. The kink-like feature disappears when a 30 kOe magnetic field is applied. Figure 4b shows the temperature-dependent specific heat in magnetic fields perpendicular to the *b*-axis. A second-order-like phase transition is observed in the field under 40 kOe. As the field increases, the transition temperature decreases. Figure 4c presents the Curie–Weiss (CW) fitting at 50 K–300 K. The CW analysis was performed on the polycrystalline average susceptibility using a magnetic field of 1 kOe, calculated as χ = (2χ_⊥_ + χ_∥_)/3 (β is close to 90 deg and the *a* and *c* lattice parameters are similar), where χ_⊥_ and χ_∥_ denote susceptibilities perpendicular and parallel to the crystallographic *b*-axis, respectively. The μ_eff_ is 7.57 μ_B_, slightly less than the value of Eu^2+^. The large positive χ_0_ may come from the Pauli contribution of the material.

**Figure 5:**
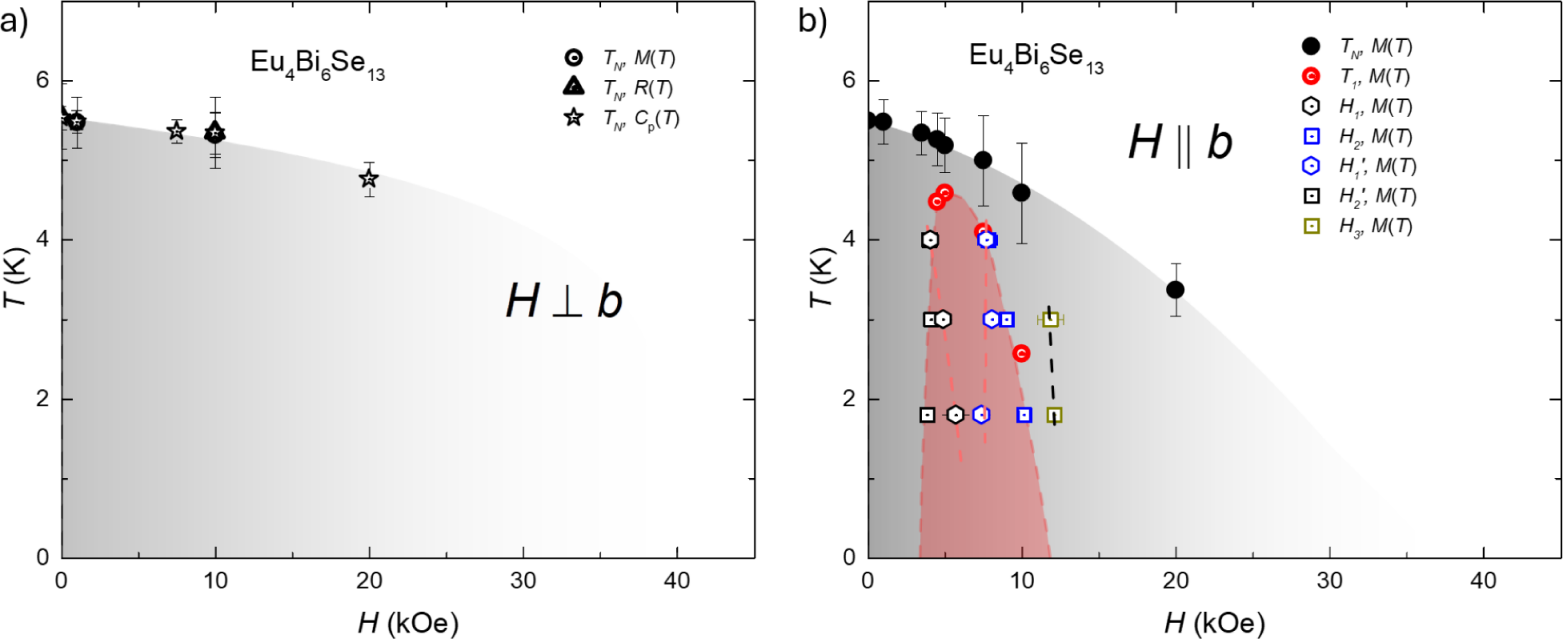
Magnetic phase diagram of Eu_4_Bi_6_Se_13_as the magnetic field perpendicular to the *b*-axis (a) or parallel to theb-axis (b). The phase diagram is plotted based on the thermodynamic and transport measurements taken on single crystals of Eu_4_Bi_6_Se_13_. The criteria of transition temperatures are shown in Figures S2a,b and S3a,b. The average of onset and offset values is transition temperature, and the transition width is determined by the half of difference of onset and offset values. The transition temperature based on temperature-dependent magnetization using *d*(*M* × *T*/*H*)*dT* is determined by round symbols. *H*_1_, *H*_2_, *H*_1_’, *H*_2_’, and *H*_3_ are determined by field-dependent magnetization measurements using *dM*/*dH* in the direction of *H* || *b*. The hollow triangle indicates the transition temperatures determined by *dR*/*dT* of temperature-dependent resistance measurement in the direction of *H* ⊥ *b*. The hollow star represents the transition temperatures based on temperature-dependent specific heat measurements. The black dashed line indicates the metamagnetic transition, and the red dashed line gives the transition in the *H* || *b* direction.

Figure 5 presents the phase diagram of Eu_4_Bi_6_Se_13_ based on the thermodynamic and transport measurements. As the magnetic field is applied perpendicular to the *b*-axis, temperature-dependent magnetization, resistance, and specific heat measurements show similar phase transitions. The transition temperatures determined by d(*M* × *T*/*H*)*dT*, *dR*/*dT,* and *C*_p_(*T*) overlap well in the phase diagrams. Considering the decrease in magnetization, the kink-like feature in resistance, and the second-order-like transition in specific heat, we suspect this transition is antiferromagnetic. The Néel temperatures, *T*_N_, as the field is perpendicular to the *b*-axis are suppressed as the magnetic field increases. In the field parallel to the *b*-axis, *T*_N_ differs from *H* ⊥ *b*. The decrease of *T*_N_ is faster as a function of the magnetic field compared with *H* || *b*. Field-dependent magnetization has more features in the *H* || *b* direction than in the other direction. As shown in Figures 3a and S2b, when the measurements were taken as the field increased, a slope change happened at *H*_1_. After that, a kink-like feature is shown at *H*_2_, corresponding to the end of hysteresis. Then, a metamagnetic feature is shown at *H*_3_ before saturation. When the measurements are taken as the field decreases, the metamagnetic feature at *H*_3_ does not change, but the magnetic fields of the other two features do change. As shown in Figure S2b, we use *H*_1_” and *H*_2_’ to represent these features. Figure 5 shows that the *T*_1_ from the temperature-dependent magnetization overlaps well with the *H*_2_ and *H*_2_’ tendencies, shown as the boundary of the red shadow. Since *T*_1_ presents the hysteresis, this may indicate that *H*_2_, *H*_2_’, and *T*_1_ correspond to the same physical phenomena: the existence of the spin-flop-induced magnetic domains due to the small slope change of moments. We suspect the appearance of magnetic domains is due to the competition among demagnetization, exchange, and magnetocrystalline energy. When the magnetic field increases, a net moment appears in the antiferromagnetic background, which may be due to spin-flop and gives the demagnetization energy. The magnetic domain appears if the demagnetization energy exceeds the domain wall’s creation energy. *H*_1_ and *H*_1_’ should correspond to the motion of the domain wall since the tendency is the same with *H*_2_ and *H*_2_’. However, the magnetic domains are not shown in the field perpendicular to the *b*-axis. This may be due to the magnetocrystalline energy, making the extra energy cost to form the domain along the field direction. *H*_3_ presents the metamagnetic transition, which does not show any hysteresis during an increase or decrease of the magnetic field.

## Conclusion

In this study, we successfully synthesized Eu_4_Bi_6_Se_13_, a metallic material, using a solid-state reaction method. This compound exhibits a monoclinic crystal structure within the *P*2_1_/*m* space group, distinguishing it from other europium bismuth chalcogenides in structural uniqueness. Notably, the material transitions into an antiferromagnetic state near 5 K and demonstrates magnetic anisotropy. The magnetic easy axis is aligned with the monoclinic axis. Magnetic characterization at low applied magnetic fields revealed metamagnetic transitions attributable to spin reorientation phenomena, likely due to the material’s inherent weak antiferromagnetic exchange relative to magnetic anisotropy. A phase diagram is plotted with a magnetic domain motion that appears under certain magnetic field and temperature ranges.

## Supporting Information Available

crystal structure refinement data; atomic coordinates and isotropic displacement parameters; PXRD refinement information at 300 K; SEM and EDS results; transition temperature values and magnetic fields; transition temperatures in resistance and specific heat; volume and β changes as a function of temperature; magnetic anisotropy of Eu_4_Bi_6_Se_13_ (PDF)
