# Supplementary material for: Spin–Flop and Metamagnetic Transition in Monoclinic Eu4Bi6Se13
Source: Chem Mater. 2025 Feb 21;37(5):1935–41. doi: 10.1021/acs.chemmater.4c03185 (PMC11905279; doi:10.1021/acs.chemmater.4c03185)

Spin-flop and Metamagnetic Transition in Monoclinic $Eu_4Bi_6Se_{13}$

Mingyu Xu[1#], *Jose L. Gonzalez Jimenez*[1#], *Greeshma C. Jose*[2], *Artittaya Boonkird*[3,4], *Chengkun Xing*[5], *Chelsea Harrod*[6], *Xinle Li*[6], *Haidong D. Zhou*[5], *Xianglin Ke*[7], *Wenli Bi*[2], *Mingda Li*[3,4], *Weiwei Xie*[1*]

1. Department of Chemistry, Michigan State University, East Lansing, MI, 48864, USA
2. Department of Physics, University of Alabama, Birmingham, AL 35294, USA
3. Quantum Measurement Group, Massachusetts Institute of Technology, Cambridge, MA, 02139, USA
4. Department of Nuclear Science and Engineering, Massachusetts Institute of Technology, Cambridge, MA, 02139, USA
5. Department of Physics, University of Tennessee, Knoxville, Tennessee 37996, USA
6. Department of Chemistry, Clark Atlanta University, Atlanta, GA 30314, USA
7. Department of Physics and Astronomy, Michigan State University, East Lansing, MI, 48864, USA

# equally contributed

Corresponding Author: Weiwei Xie (xieweiwe@msu.edu)

Table S1. Crystal structure refinement data……………………………………………………S2

Table S2. Atomic coordinates and isotropic displacement parameters……...............................S3

Table S3. PXRD refinement information at 300 K………...S4

Table S4. Anisotropic displacement parameters ………..S4

Figure S1. SEM and EDS results…………………………………………………………..S5

Figure S2. Transition temperature values and magnetic fields …………………………………….S6

Figure S3. Transition temperatures in resistance and specific heat ……………………………………S7

Figure S4. Volume and β changes as a function of temperature …………………………………….…S8

Figure S5. Magnetic anisotropy of $Eu_4Bi_6Se_{13}$……………………………………………….S9

Table S1. Single crystal crystallographic data and structure refinement for $Eu_4Bi_6Se_{13}$ single crystal at 100 K. Values in parentheses are estimated standard deviations from refinement.

**Refined Formula**	**$Eu_4Bi_6Se_{13}$**
F. W. (g/mol)	2888.20
Temperature (K)	100.15
Space group	$P2_1/m$
a (Å)	16.8965(2)
b (Å)	4.2194(5)
c (Å)	18.2479(2)
β (°)	90.6664(12)
V (Å^3)	1300.87(3)
Z	2
2θ range (°)	5.05 to 84.534
Density (calculated)	7.373 g/cm^3
Absorption coefficient	68.082 mm^{-1}
Reflections collected	118175
Independent reflections	9991 [R_{int} = 0.0822]
Refinement method	Full-matrix least-squares on F^2
Data/restraints/parameters	9991/0/139
Final R indices	R_1 (I>2σ(I)) = 0.0280; wR_2 (I > 2 σ(I)) = 0.0534<br>R_1 (all) = 0.0393; wR_2 (all) = 0.0564
Largest diff. peak and hole	+3.69 e^-/Å^3 and -4.48 e^-/Å^3
R. M. S. deviation from mean	0.606 e^-/Å^3
Goodness of fit	1.044

Table S2. Atomic coordinates and isotropic displacement parameters of $Eu_4Bi_6Se_{13}$ single crystal. (U_{eq} is defined as one-third of the trace of the orthogonalized U_{ij} tensor.) Values in parentheses are estimated standard deviations from refinement.

Atom	Wyck.	Occ.	*x*	*y*	*z*	U_{eq}
Eu1	2*e*	1	0.01202(2)	1/4	0.75210(2)	0.00486(4)
Eu2	2*e*	1	0.27071(2)	1/4	0.82146(2)	0.00537(4)
Eu3	2*e*	1	0.48394(2)	1/4	0.65651(2)	0.00598(4)
Eu4	2*e*	1	0.74374(2)	1/4	0.73077(2)	0.00542(4)
Bi1	2*e*	1	0.08313(2)	1/4	0.42140(2)	0.00547(3)
Bi2	2*e*	1	0.82477(2)	1/4	0.38014(2)	0.00531(3)
Bi3	2*e*	1	0.32919(2)	1/4	0.47713(2)	0.00438(3)
Bi4	2*e*	1	0.50754(2)	1/4	0.12273(2)	0.00523(3)
Bi5	2*e*	1	0.68542(2)	1/4	0.95803(2)	0.00587(3)
Bi6	2*e*	1	0.10763(2)	1/4	0.02382(2)	0.00894(4)
Se1	2*e*	1	0.37666(3)	1/4	0.33475(3)	0.00467(8)
Se2	2*e*	1	0.80043(3)	1/4	0.55944(3)	0.00549(8)
Se3	2*e*	1	0.04141(3)	1/4	0.59118(3)	0.00501(8)
Se4	2*e*	1	0.56897(3)	1/4	0.48450(3)	0.00518(8)
Se5	2*e*	1	0.97929(4)	1/4	0.91020(3)	0.00949(9)
Se6	2*e*	1	0.23691(3)	1/4	0.12903(3)	0.00628(8)
Se7	2*e*	1	0.85999(3)	1/4	0.23489(3)	0.00657(8)
Se8	2*e*	1	0.60715(3)	1/4	0.23850(3)	0.00490(8)
Se9	2*e*	1	0.39453(3)	1/4	0.95888(3)	0.00589(8)
Se10	2*e*	1	0.11435(3)	1/4	0.27469(3)	0.00606(8)
Se11	2*e*	1	0.57888(3)	1/4	0.81538(3)	0.00510(8)
Se12	2*e*	1	0.28187(3)	1/4	0.64428(3)	0.00524(8)
Se13	2*e*	1	0.80370(3)	1/4	0.06214(3)	0.00663(8)

The structure was solved and refined using the Bruker SHELXTL Software Package, using the space group $P2_1/m$, with Z = 2 for the formula unit $Eu_4Bi_6Se_{13}$. The final anisotropic full-matrix least-squares refinement on F^2 with 139 variables converged at R_1 = 3.93 % for the observed data and wR_2 = 5.64 % for all data. The goodness-of-fit was 1.044. The largest peak in the final difference electron density synthesis was 3.69 $e^-/Å^3$, and the largest hole was -4.48 $e^-/Å^3$ with an RMS deviation of 0.606 $e^-/Å^3$. Based on the final model, the calculated density was 7.373 g/cm^3 and F (000), 2384 e^-.

Table S3. PXRD refinement information at 300 K.

**Refined Formula**	**$Eu_4Bi_6Se_{13}$**
F. W. (g/mol)	2888.20
Temperature (K)	300
Pressure (GPa)	0.37
Space group	$P2_1/m$
a (Å)	16.94947
b (Å)	4.226467
c (Å)	18.279269
β (°)	90.5899
V (Å^3)	1309.391
Z	2
2θ range (º)	1.60 to 16.00
Density (calculated)	7.325 g/cm^3
wR	4.625

Table S4. Anisotropic displacement parameters.(Å^2×10^3)

Atom	U_{11}	U_{22}	U_{33}
Eu1	3.50(9)	6.42(9)	4.65(9)
Eu2	5.28(10)	5.59(9)	5.24(9)
Eu3	5.6(1)	5.80(9)	6.52(10)
Eu4	4.89(10)	5.70(9)	5.68(9)
Bi1	5.03(7)	6.24(7)	5.15(7)
Bi2	5.29(7)	5.98(7)	4.65(7)
Bi3	3.85(7)	5.07(7)	4.22(6)
Bi4	4.96(7)	6.14(7)	4.59(7)
Bi5	5.15(7)	7.44(7)	5.01(7)
Bi6	7.14(8)	7.94(7)	11.75(8)
Se1	4.5(2)	5.80(19)	3.70(18)
Se2	4.0(2)	6.24(19)	6.2(2)
Se3	4.5(2)	6.52(19)	4.03(19)
Se4	4.0(2)	5.51(19)	5.97(19)
Se5	8.2(2)	9.7(2)	10.6(2)
Se6	6.6(2)	7.7(2)	4.65(19)
Se7	6.6(2)	8.9(2)	4.25(19)
Se8	4.6(2)	5.73(19)	4.40(18)
Se9	6.0(2)	6.14(19)	5.62(19)
Se10	6.3(2)	7.4(2)	4.47(19)
Se11	4.4(2)	6.00(19)	4.94(19)
Se12	4.5(2)	6.02(19)	5.24(19)
Se13	6.6(2)	7.8(2)	5.4(2)

Figure S1. SEM and EDS results of single crystal $Eu_4Bi_6Se_{13}$. The picture shows the SEM result of the sample, and the table summarizes the EDS results of several spots from two different samples.

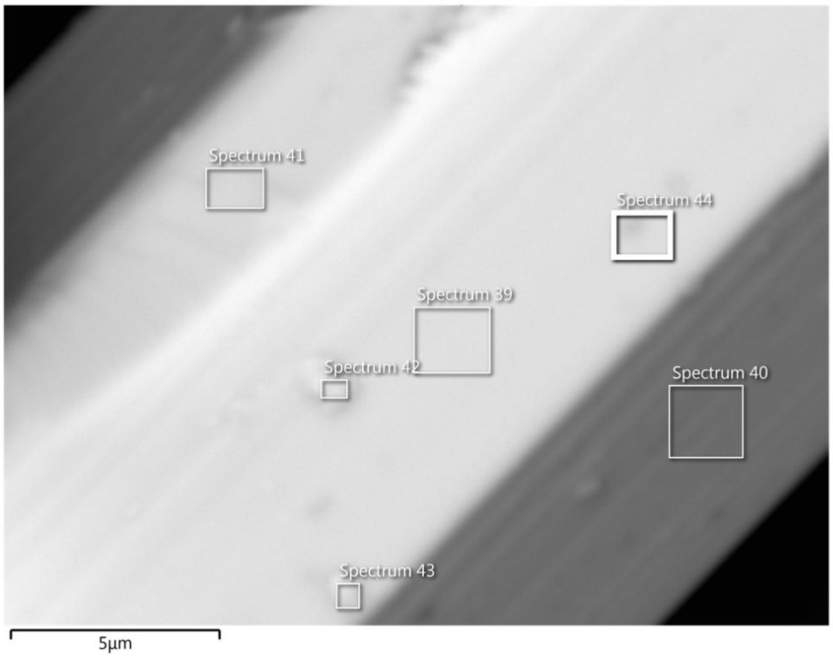

Elements	At%
Eu	16(1)
Bi	28(1)
Se	56(2)

Figure S2. Onset and offset values of transition temperatures and fields of temperature-dependent (Fig. S2*a*) and field-dependent (Fig. S2*b*) magnetization. The criteria of feature temperatures and fields are defined using onset and offset values. The transition temperature is defined as the average value of onset and offset, and the transition width is defined as half of the difference between the two values (shown as the error bar in the phase diagram). The inset of **Fig. S2*a*** gives the criterion of T_1, which is decided by the cross of two lines. The two straight lines try to fit the magnetization curve near the crossing. As shown in **Fig. S2*b***, since the existence of hysteresis, the feature fields are defined separately by magnetization measured as the field increases (black) and decreases (red). Since the dM/dH data of field decrease is shifted by a constant to separate the features, the original dM/dH plot is shown in the inset.

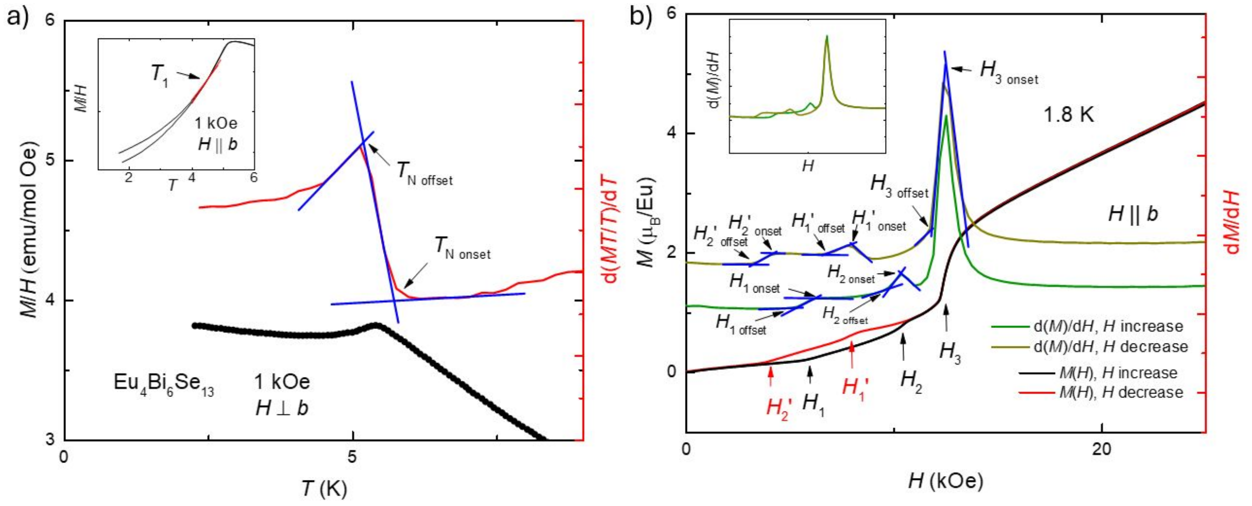

Figure S3. Onset and offset values of transition temperatures of temperature-dependent resistance (Fig. S3*a*) and specific heat (Fig. S3*b*). The criteria of feature temperatures are defined using onset and offset values. The transition temperature is defined as the average value of onset and offset, and the transition width is defined as half of the difference between the two values (shown as the error bar in the phase diagram).

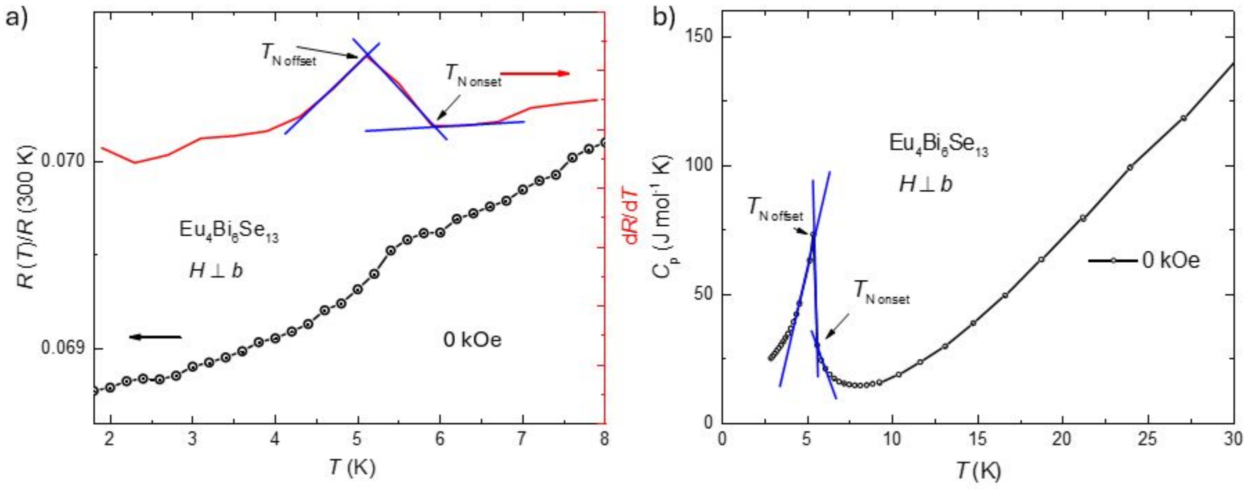

Figure S4. Volume and β changes as a function of temperature

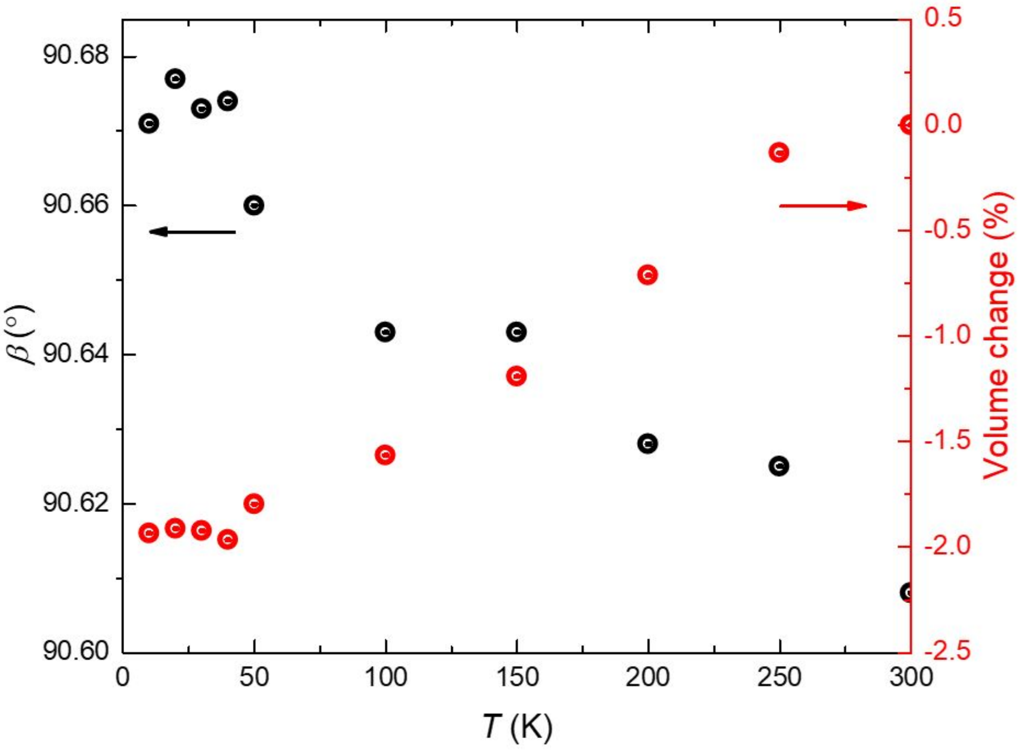

Figure S5. Magnetic anisotropy of $Eu_4Bi_6Se_{13}$

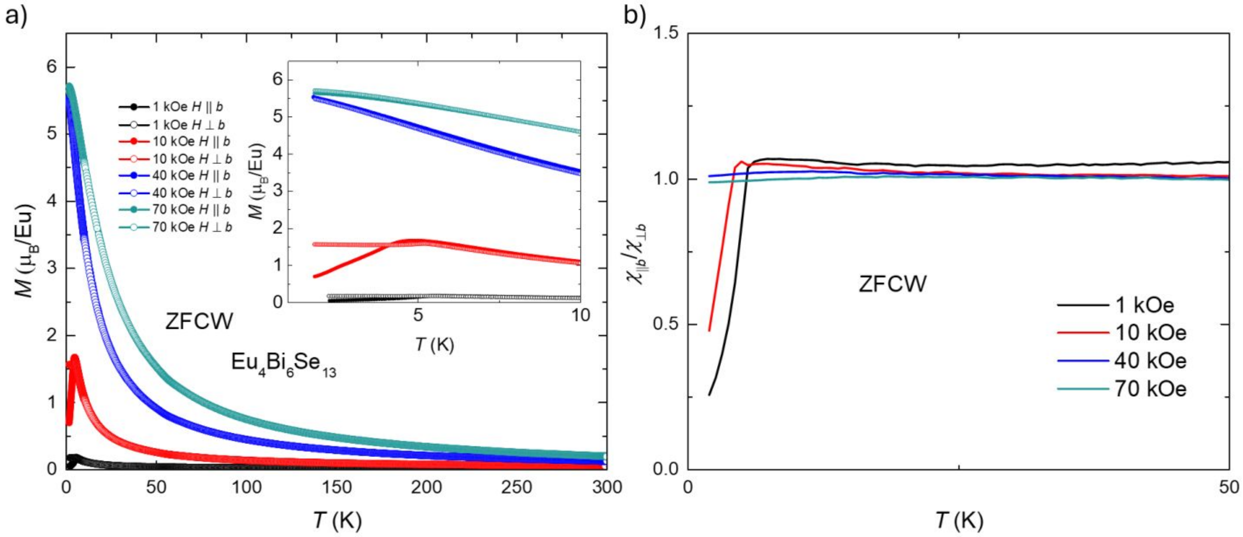

Supplement: Supplementary file 1 — cm4c03185_si_001.pdf [file cm4c03185_si_001.pdf]
